# How to implement environmental sustainability in the OR in gynaecology: to measure is to know

**DOI:** 10.52054/FVVO.14.1.012

**Published:** 2022-04-03

**Authors:** K.E. Van Nieuwenhuizen, F.W. Jansen

**Affiliations:** Department of Gynaecology, Leiden University Medical Centre, 2333 ZA, Leiden, the Netherlands; Department of Biomechanical Engineering, Delft University of Technology, 2628 CD, Delft, the Netherlands

Climate change, as a result of global warming, inherently threatens human health. The visible changes in temperature and precipitation and thereby more frequent extreme weather conditions affect human health unmistakably. (Watts et al., 2018) Global healthcare has an environmental impact that varies between 1-5% of the total impact worldwide. (Lenzen et al., 2020) For some national impacts this accounts for more than 5%, even increasing to 11% of the total national environmental impact. Therefore, the paradox is, that healthcare is partly responsible for the negative effects on public health through, among other things, CO_2_ emissions and depletion of raw materials (Eckelman and Sherman, 2018).

At the 26^th^ UN Climate Change Conference of the Parties (COP26) in Glasgow, the importance of the scientific conclusions of the Intergovernmental Panel on Climate Change (IPCC) emphasized that action is mandatory. (Masson-Delmotte et al., 2021) As medical professionals we have to take responsibility, climate change is happening and we have to act now. In the United Kingdom, the National Health Service (NHS) has already reduced its carbon footprint with 11% between 2007 and 2015 by taking measures on the three aforementioned points (SD Unit, 2015).

International and Dutch climate policy is currently aimed to reduce the national CO_2_ emissions by 49% in 2030 and 95% in 2050 compared to the reference year 1990. To achieve this goal, an annual reduction of 6 to 8% is necessary. (Gupta Strategists, 2019) For the Dutch healthcare sector, this ambition has been captured in 2018 in the governmental pushed Greendeal 2.0 ‘Sustainable care for a healthy future’ (Greendeal 2.0. https://milieuplatformzorg.nl/green-deal/).

In order to achieve CO_2_ -neutral healthcare in 2050, it is important that measures are sought throughout the sector that are most effective in terms of impact and feasibility. For example, it is known that the operating room (OR) has a high energy consumption and a high consumption of goods and therefore generates a lot of waste. (MacNeill et al., 2017) It is estimated that 20 to 30% of the total hospital waste comes from the OR. (Axelrod et al., 2014) Especially in the OR certain measures can be taken to reduce CO_2_ emission, in order to contribute in reducing CO_2_ emissions for the whole hospital. In addition, measures in the nursing wards can be conducive to that CO_2_ reduction. Unfortunately, it is not easy to know where to start and to what extent certain measures contribute to the reduction of waste. In short, ‘to measure is to know’.

The CO_2_ footprint (in CO_2_ -equivalents) expresses the amount of greenhouse gases emitted by a sector, company or individual. This can be directly emitted greenhouse gases, such as CO_2_ due to the combustion of fossil fuels or the emission of inhalation anaesthetics, but also indirect emissions, for example those released during the generation of purchased electricity or heat. Another important source of indirect greenhouse gas emissions originates from the production of goods and processing of waste. The total environmental impact during the life cycle of products or services can be calculated by the means of a Life Cycle Assessment (LCA). (Guinée et al. 2011) Although in general a lot has to be done to reduce this impact, healthcare hides action due to patient safety matters. However, in close collaboration with engineers from the Delft University of Technology (TUD) and the Faculty of Industrial Ecology of Leiden University, a number of projects in the OR of the Leiden University Medical Centre (LUMC) has been set up, to gain insight through quantification where and how we can reduce our environmental impact.

## CO_2_ reduction in OR centre and central sterile services department (CSSD)

In the OR, the main contributors in CO_2_ emission are inhalation anaesthetics, energy consumption through air treatment and the procurement of goods and waste production. (MacNeill et al. 2017) Although the indication and use of inhalation anaesthetics is in hands of the anaesthesiologist, the use of gases can be reduced by switching from inhalation anaesthesia (e.g. Desflurane or Sevoflurane) to intravenous anaesthesia (Propofol). By the change towards preferably the use of intravenous anaesthetics, this component is no longer a significant contributor to the CO_2_ footprint at the OR in the LUMC.

In order to map the CO_2_ footprint of the OR, the CO_2_ emission hotspots were identified with a quick scan. This showed that, the OR in the LUMC emitted 1500 tons of CO_2_ -equivalents in the year 2020. The emission of inhalation anaesthetics led to 35 tons of CO_2_ -equivalents, the air treatment produced 715 tons of CO_2_ -equivalents and the waste accounted for 750 tons of CO_2_ -equivalents per year. As a comparison: one passenger car which travels 20,000 kilometres (km) per year produces about 3.5 tons of CO_2_ -equivalents.

In order to further develop the principle of ‘to measure is to know’, a special sustainable OR was set up in the LUMC. In this OR research to reduce the CO_2_ footprint is conducted. A method has been developed that directly links sustainable solutions to the CO_2_ footprint. This measurement method, modified according to the Healthcare Failure Mode and Effect Analysis (HFMEA) principle (van den Haak et al., 2018) was introduced as the HSMEA (Healthcare Sustainability Mode and Effect Analysis). (unpublished data) This calculation method makes it possible to compare different sustainable solutions in daily practice and determine which solutions provide the greatest CO_2_ reduction. In this way, priority can be given to the ‘most sustainable’ initiatives, in terms of CO_2_ emissions. Specifically, this HSMEA was applied to the waste production of a caesarean section (CS). It showed that a CO_2_ reduction of 24% can be achieved. To achieve this, the clean packaging waste in the refinishing area must already be separated into streams of plastic, paper and residual waste. However, this only reduces CO_2_ emissions by about 1%. A much greater CO_2_ reduction can be achieved by optimizing the procedure tray for the CS. Of this procedure tray, 22% was not used or not usefully used. However, the standard removal of things from the procedure tray has to be completed by the manufacturer. In this way, waste for a CS can be reduced by 23%. Similar results have also been found in other studies (Huncke et al., 2012; Campion et al., 2015).

Another process which is very energy and labour intensive is the sterilization process. It is shown that a large part of the surgical instruments, available on the instrument tray, are not used during an operation. Just like used instruments, these ‘clean instruments’ are cleaned and sterilized again after surgery. Unnecessary instruments on the surgical tray are thus a good source for cost reduction and removing them (indirectly) improves efficiency and contributes in reducing CO_2_ emissions. For example, a method has recently been developed to measure how the number of instruments on a surgical tray can be reduced effectively. (unpublished data) With this method, initially by manually keeping track of which instruments are actually used per operation, a reduction of instruments could be determined on each surgical tray. For the gynaecological OR tray, this resulted in a reduction of 40%. After both surgeons and operating room assistants agreed the measured reduction on the surgical tray was feasible, the method was validated and it can now be used in the entire operating room complex. Although the total reduction potential of the surgical trays is still unknown at this time, the average reduction percentage will have a significant impact on the amount of energy, electricity, chemicals, water, steam and the environmental impact associated with the washing and disinfection process of the instruments. In a hospital-wide implementation, the capacity of the cleaning and sterilization equipment can most likely be reduced by about 30%, with a corresponding reduction in costs and CO_2_ footprint. Since the staff of the sterilization department, who account for about half of the total costs of the department, spend a lot of time organizing and inspecting each individual instrument, this will not only improve efficiency, but also indirectly achieve CO_2_ reduction (unpublished data).

The use of single-use packaging materials for sterile surgical nets (the so-called blue wrap) has also been shown to produce a lot of waste and CO_2_ , compared to the globally widely used reusable containers for sterilization nets. The analysis, performed at the LUMC, shows that when the reusable container is used for twenty sterilization procedures, environmental benefits are already achieved compared to single-use packaging material.

Furthermore, the use of reusable OR gowns in comparison to disposable OR gowns leads to a lower environmental impact. (Vozzola et al., 2020; Overcash 2012) Since, next to the risk of contamination, comfort was also a factor of the change towards disposable OR gowns, it is interesting to see whether reusable OR gowns can still have a place in the OR. With regard to comfort, reusable surgical gowns have developed over the past years, however the last study on comfort of disposable and reusable surgical gowns was conducted in 2010 (Conrardy et al., 2010). Recently, a study has been conducted at our sustainable OR to compare the comfort of reusable and disposable sterile OR gowns. The majority of the users score the comfort of the reusable OR gown as equal or better in comparison to the disposable OR gown. With this knowledge, a shift towards reusable sterile OR gowns will benefit in reducing the CO_2_ footprint of the OR.

## What are the next steps in reducing the CO_2_ footprint?

In addition to the statement ‘to measure is to know’, awareness about this subject has turned out to be a conditio sine qua non. Unfortunately, much is still unknown and many people do not realize that measures can be taken in the hospital workplace that contribute to a reduction of the CO_2_ footprint. (Friedericy et al. 2019) As doctors, we must create awareness for the consequences of our medical-ecological actions. Not only in the workplace, but certainly also with the next generation of healthcare professionals. By teach- ing knowledge about sustainability in healthcare in the courses, it will become part of the competences of the next generation of doctors and nurses. The Environmental Care Platform is currently developing roadmaps that can serve as a guideline for making care more sustainable. (MilieuplatformZorg)

In the LUMC, so-called ‘Green Teams’ have been set up in various departments, which explore where benefits can be achieved from an environmental point of view, according to a centrally supplied format. Not only the separation of waste, but also far-reaching actions are taken to actually reduce the CO_2_ foot- print. For example, at the moment waste is not only separated in the OR complex, but the tableware has been replaced by compostable plates, cutlery and cups and the (expensive) drinking water bottles have been replaced by a water tap. In addition, a study has been launched to achieve waste reduction by ex- amining how reusable instruments and materials can be used instead of disposable instruments. Thereby, recycling can prevent raw materials from being lost.

In the near future, the impact on the environment will also have to play a role in the treatment of patients. Although this is an ethical issue as difficult as that of the cost of a treatment, the treating physi- cian will have to consider the environmental interest in addition to the interest of the individual patient. Naturally, the quality and safety of patient care is paramount. A good example in this regard is a recent US study of the CO_2_ footprint of the hysterectomy (vaginal versus abdominal versus laparoscopic or robot-assisted). Here it was found that the robot-assisted hysterectomy generates as much as 60% more waste than the (least polluting) vaginal hysterectomy. (Thiel et al. 2015)

## Act immediately

What can I do tomorrow to make my OR more sustainable?

1. Inventory whether the circulating air (laminar flow) in the OR can be switched off at night. By letting this laminar flow only work when the ORs are in use, a saving on energy consumption of 50% is possible. In case of emergency interventions, the quality is sufficient within 30 minutes after start-up. It is also possible to choose to have one or more emergency ORs always ready for operating at night.
2. Ask the anaesthesiologists not to use Desflurane and nitrous oxide in any case and to use as little Sevoflurane as possible. Intravenous and regional anaesthesia have a negligible carbon footprint compared to inhalation anaesthetics.
3. It is also important that the amount of disposables is reduced. Almost all LCAs show that reusables are less harmful to the environment than disposables. This applies to sterile surgical gowns, but also instruments such as scissors, tweezers and coagulation instruments.
4. Waste separation and recycling in the OR is possible, provided there is no risk of infection. This means that opportunities are created for this in the coffee rooms and other areas.
5. Setting up an OR Green Team is recommended to create awareness, to collect initiatives and to honour them.

## Consideration

The ambition, as laid down in the Green Deal 2.0, to contribute with the healthcare sector to achieve the target of a CO_2_ reduction of 95% by 2050 is a good intention. However, only with the principle of ‘to measure is to know’ this plan will get real. With the help of technicians (in this case from the TUD and the Faculty of Industrial Ecology of Leiden University), we have been able to provide quantitative and effective insight into the CO_2_ footprint and to know where effective reductions can be achieved. A lot can be achieved with the principle of the 10Rs of circularity: Refuse, Reduce, Repair, Refurbish, Remanufac- ture, Re-purpose, Redesign, Recycle, Recover (energy). However, the most important R in reducing our CO_2_ footprint is: Refuse, or think before you start. The use of inhalation anaesthetics and the application of robotic surgery, on non-strict indication, are examples of this. In order to guarantee the quality of life on our planet, we as healthcare professionals, will have to take responsibility for taking measures in our own domain that guarantee both patient safety and the environment’s. (Friedericy et al. 2019) With the help of third parties (including engineers), ‘to measure is to know’ can give shape and substance to this pursuit and let us steam up to a permanently liveable earth for next generations.
